# Interprofessional contact with conventional healthcare providers in oncology: a survey among complementary medicine practitioners

**DOI:** 10.1186/s12906-024-04563-6

**Published:** 2024-07-26

**Authors:** Marit Mentink, Julia Jansen, Janneke Noordman, Liesbeth van Vliet, Martine Busch, Sandra van Dulmen

**Affiliations:** 1 Department of Communication in Healthcare, Nivel, Netherlands institute for health services research, Utrecht, the Netherlands; 2 Radboud Institute for Health Sciences, Department of Primary and Community Care, Radboud university medical center, Nijmegen, The Netherlands; 3 Health, Medical and Neuropsychology Unit, Institute of Psychology, Leiden University, Leiden, The Netherlands; 4 Leiden Institute for Brain and Cognition, Leiden University, Leiden, The Netherlands; 5 Van Praag Institute, Utrecht, Netherlands; 6 Faculty of Caring Science, University of Borås, Work Life and Social Welfare, Borås, Sweden

**Keywords:** Complementary medicine, Interprofessional contact, Complementary medicine practitioner, Healthcare provider, Healthcare communication, Oncology, Cancer

## Abstract

**Background:**

Half of all patients with cancer use complementary medicine. Given the benefits and risks associated with complementary medicine use, contact between complementary medicine practitioners and conventional healthcare providers (oncologists, nurses) is important for monitoring the health and well-being of mutual patients with cancer. Research on occurrence of such interprofessional contact is scarce. This study aims to describe complementary medicine practitioners’ experiences with contact with conventional healthcare providers about mutual patients with cancer and the importance they attach to patient disclosure of complementary medicine use to their conventional healthcare provider. Predictors for interprofessional contact are explored.

**Methods:**

An online survey was administered among complementary medicine practitioners who treat patients with cancer or cancer survivors and who are member of a professional association in the Netherlands.

**Results:**

The survey was completed by 1481 complementary medicine practitioners. 40% of the participants reported to have contact with conventional healthcare providers of patients with cancer. Only 13% of the complementary medicine practitioners experienced conventional healthcare providers as open to communication with them. An explorative logistic regression showed that openness of conventional healthcare providers as experienced by complementary medicine practitioners was the most important predictor for the occurrence of interprofessional contact (OR = 8.12, 95% CI 5.12–12.86, *p* < .001). Most complementary medicine practitioners (82%) considered it important that patients disclose complementary medicine use to their conventional healthcare provider and 49% of the participants always motivates their patients to do so.

**Conclusions:**

Interprofessional contact with conventional healthcare providers in oncology occurs but is not routine for most complementary medicine practitioners. More than one-third of the surveyed complementary medicine practitioners experienced conventional healthcare providers as not open to communication with them. The openness of conventional healthcare providers as experienced by complementary practitioners is an important predictor for interprofessional contact to take place. Most complementary practitioners acknowledge the importance of patient disclosure of complementary medicine use to their conventional healthcare provider. Open communication about the topic of complementary medicine use between complementary practitioners, conventional healthcare providers and patients prevents overlooking relevant medical information and facilitates optimal monitoring of health and safety of patients with cancer.

**Supplementary Information:**

The online version contains supplementary material available at 10.1186/s12906-024-04563-6.

## Background

Approximately half of all patients with cancer use complementary medicine (CM) [1]. CM is a healthcare approach that is being used alongside conventional cancer treatment and includes many therapies, such as massage, acupuncture and nutritional supplements [2]. CM can benefit the quality of life of patients with cancer, for instance acupuncture can be used for cancer pain management [3] and mindfulness-based interventions for depression and anxiety during cancer treatment [4]. However, CM can also pose a risk to patients with cancer, for example when herbs and supplements interact with chemotherapy [5].

Given the potential benefits and risks for patients with cancer that use CM, communication between individuals providing CM (CM practitioners) and conventional healthcare providers (HCPs) is important for monitoring the health and safety of patients with cancer. However, there seem to be several barriers to such interprofessional contact. Generally, CM practitioners are located outside the hospital and often work independently of conventional HCPs such as oncologists and nurses. Other barriers described in two previous studies were unfamiliarity with each other’s medical system, language barriers due to distinct terminology [6], medical dominance of conventional HCPs and the lack of role clarity [7]. There are no guidelines available on interprofessional communication about CM between CM practitioners and conventional HCPs.

A previous study showed that physicians and CM practitioners regarded communication with each other as important, although only 7% of physicians and 18% of CM practitioners reported previously having such interprofessional contact [6]. Importantly, only one previous study was conducted in an oncology setting and assessed actions to improve communication between CM practitioners and conventional HCPs in oncology, such as being trained in the other field, using common medical terminology and being located in the same practice [8]. To the best of our knowledge, no further studies have been conducted on contact between CM practitioners and conventional HCPs about mutual patients with cancer.

Additionally, previous research shows that many patients with cancer do not disclose their CM use to their conventional HCP for reasons such as lack of inquiry or anticipated disapproval [9]. The potential role of CM practitioners in motivating disclosure of CM use by patients to their conventional HCPs remains unclear.

This study therefore aims to assess CM practitioners’ experiences with interprofessional contact with conventional HCPs about mutual patients with cancer and the importance they attach to patient disclosure of CM use to their conventional HCP. Potential predictors for interprofessional contact will be explored.

## Methods

An online survey was administered among complementary medicine (CM) practitioners in the Netherlands. This study is part of a larger mixed-method research project titled ‘COMMON’ [10].

### Participants and sampling

CM practitioners were eligible for participation if they (1) currently treated patients with cancer or cancer survivors and (2) were members of a professional association for CM practitioners. Membership in a professional association is an important quality criterion for CM practitioners in the Netherlands [11]. To recruit participants, a combination of convenience and purposive sampling was used. Eight professional associations of CM practitioners were directly approached with the request to distribute a link to the online version of the survey among their members. One association did not respond to the request, seven associations agreed with distributing the survey link (see Additional file 1, Table A1). The largest participating association (*n* = 8858) was the Register for Complementary Medicine (RBCZ), an umbrella quality register for complementary medicine practitioners in the Netherlands. In addition, RBCZ requested 24 attached professional associations to distribute the link among their members (e.g. Dutch associations for naturopathy, psychology, homeopathy, shiatsu and reflexology). In response to the distributed survey link, two professional organizations approached us with the request to distribute the survey link among their members (i.e. snowball sampling). The average response rate among the seven actively approached professional associations was 9%. The number of members at time of survey administration of members attached to other associations is unknown, so a response rate could not be calculated.

### Materials and measures

The survey was designed by the research team. First, the researchers (SvD, JJ, MB) defined important themes in a brainstorm session and subsequently created a first draft of the survey. This draft was piloted in a group of coresearchers, consisting of nine (former) patients with cancer. The improvements based on this pilot consisted of the addition of answer options for three survey questions and minor adjustments in sentencing to improve comprehensibility of the questions or answer options. The final survey consisted of 17 items, including both open-ended and closed questions (see Additional file 2 for full survey). The first 10 items consisted of background characteristics of CM practitioners, such as demographics and the type of CM they provide to patients with cancer. To assess CM practitioner experiences with interprofessional contact, four items were included (e.g. contact frequency with conventional HCPs, experienced openness of conventional HCPs to communication). Two items consider the importance attached to patient disclosure about CM use. Last, a question about referral of patients with cancer to the CM practitioner was included. A link was created to direct participants to an online version of the survey. When For statistical analysis, SPSS version 27 was used.

### Data collection and analysis

When opening the survey link, participants were first provided with information about the study, for instance about data use and expected time for survey completion (10–15 min). Participants were then asked to sign an online informed consent form and background characteristics were collected. If participants indicated that they did not treat patients with cancer or cancer survivors, they were thanked for their participation and excluded from the rest of the survey. The link to the online survey remained open for 2 months (Aug-Sep 2022). In the first week of September 2022, the approached participating professional organizations sent a reminder to their members about the survey.

After finishing data collection, one researcher (MM) recoded the answers to open questions into relevant categories using qualitative analysis. Because of the large amount of categories for type of cancer of visiting patients, type of CM modality provided and type of symptom treated, only the five most common categories were reported in the Results section. Question 11 (“When you provide therapy to patients who have/had cancer, in general how often do you have contact with doctors or nurses who treat the patient?”) was recoded into three categories. The first category (‘no’) consisted of participants who indicated that they never have contact with conventional HCPs about their mutual patients with cancer. The second category (‘yes’) comprised participants who indicated to have contact with conventional HCPs during patient treatment, independent of the contact frequency. Answers that did not fit into these two categories (e.g. contact only through patients) were categorized as ‘other’. It was decided to exclude question 17 (“How do patients who have/had cancer get to visit you?”) from analysis because its answer categories were not mutually exclusive and the word ‘referral’ was not clearly defined in the answer options.

Descriptive statistics were used to present the data on background characteristics, experiences of CM practitioners with interprofessional contact and the importance they attach to patient disclosure of CM use. To explore factors that predict contact between CM practitioners and conventional HCPs, a logistic regression analysis (two-sided, *p* < .05) was performed in consultation with a statistician. The dependent variable ‘interprofessional contact’ (Q11) was recoded into a binary variable (yes/no) by excluding the ‘other’ category. Of the available variables, six seemed relevant and appropriate as predictor. The predictor ‘sex’ (Q2) was also recoded into a binary variable (male/female) by removing the category ‘other’, which consisted of only four participants. For each predictor, the largest category was used as a reference.

## Results

**Fig. 1:**
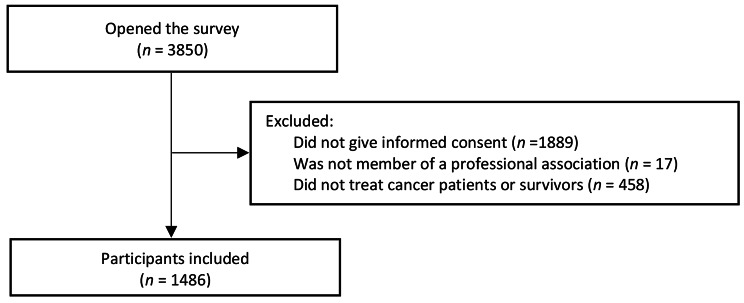
Flowchart exclusion criteria survey response

In total, 1961 participants gave informed consent for participation, of which 17 participants were excluded because they were not members of a professional association (see Figs. 1) and 458 participants because they indicated that they did not treat patients with cancer or cancer survivors. Eventually, 1486 participants were included.

Most participating CM practitioners were female (82%), with a mean age of 56.9 years (SD = 8.1) (see Table 1). Years of experience treating patients with cancer ranged from 0 to 45 years, with a mean of 11.4 years (SD = 8.5). On average, CM practitioners reported being visited by 3 to 4 patients with cancer per month.

**Table 1:** Background characteristics of surveyed complementary medicine practitioners (*n* = 1486)

Variable	No. (%)
Sex	
Male	255 (17%)
Female	1219 (82%)
Other	4 (0%)
missing	8 (1%)
Age in years	
Mean (SD)	56.9 (8.1)
Range	26–84
missing	17 (1%)
Education level ^a^	
Low	0 (0%)
Intermediate	6 (0%)
High	1454 (98%)
missing	26 (2%)
Mean years of experience with treating cancer patients	
Mean (SD)	11.4 (8.5)
Range	0–45
missing	46 (3%)
Mean monthly number of cancer patient visiting	
Mean (SD)	3.5 (6.9)
Range	0-124
missing	90 (6%)
Five most common cancer types of patients visiting CM practitioners ^b^	
Breast	692 (47%)
Colorectal	414 (28%)
Blood	296 (20%)
Lung	250 (17%)
Prostate	192 (13%)
Five most provided complementary therapies by CM practitioners ^b^	
Massage therapy	479 (32%)
Lifestyle counselling	439 (30%)
Relaxation exercises	418 (28%)
Dietary counselling	340 (29%)
Acupuncture	310 (21%)
Five most treated cancer patient symptoms by CM practitioners ^b^	
Fatigue	1240 (83%)
Anxiety	1012 (68%)
Sleeping problems	944 (64%)
Depression	874 (59%)
Concentration problems	684 (46%)

Percentages may add to less or more than 100% due to rounding

^a^ Classified according to CBS 2017 [12]

^b^ Participants could choose multiple answer options. See Additional file 1 for a comprehensive overview

### Experiences with interprofessional contact

Half of the surveyed CM practitioners indicated that they do not have contact with conventional HCPs (see Table 2). 40% of the participants had occasional or frequent contact with conventional HCPs of patients with cancer. CM practitioners who gave other answers for instance indicated that contact with the conventional HCP only takes place through the patient. More than one-third of the CM practitioners (35%) did not experience conventional HCPs to be open to interprofessional communication.

If CM practitioners communicated with conventional HCPs, this was most frequently by phone (36%). CM practitioners reached out to conventional HCPs to report the treatment plan (27%) or treatment progress (32%). This was sometimes preceded by a referral from a conventional HCP, as appeared from the answers to this open-ended question. In many cases, respondents mentioned that they do not receive a response from the conventional HCP to their report. In other cases (21%), contact between CM practitioners and conventional HCPs consisted of joint coordination, for instance by discussing contraindications for CM use.

### Importance of patient disclosure of CM use

The majority (82%) of the CM practitioners indicated that they consider it important that patients disclose their CM use to their conventional HCP and that approximately half of the CM practitioners always motivate their patients to do so. CM practitioners who gave other answers frequently mentioned that patients were anxious to disclose CM use to their conventional HCP.

**Table 2:** Experiences with contact with conventional healthcare providers (HCPs) and importance of patient disclosure of CM use as reported by complementary medicine (CM) practitioners (*n* = 1486)

Variable							No. missing (%)
**Do you have contact with conventional HCPs of patients with cancer?**							
	Yes	No	Other ^a^				
	591 (40%)	743 (50%)	133 (9%)				19 (1%)
**If yes, which method do you use for communication with conventional HCP? (** *n* ** = 591)**							
	Phone	E-mail	Letter	Electronic medical record	Face to face	Other ^b^	
	212 (36%)	116 (20%)	96 (16%)	26 (4%)	20 (3%)	114 (19%)	7 (1%)
**If yes, what is the subject of communication with conventional HCP (** *n* ** = 591)**							
	To report progress or evaluation of complementary treatment	To report complementary treatment plan	To align conventional and complementary treatment	To ask for or share patient information	Other ^c^		
	187 (32%)	158 (27%)	126 (21%)	86 (15%)	83 (14%)		55 (9%)
**How do you experience conventional HCPs’ openness to communication about patients with cancer?**							
	Most clinicians are not open	Most clinicians are open	No opinion	Other ^d^			
	525 (35%)	193 (13%)	427 (29%)	327 (22%)			14 (1%)
**How important do you think it is that patients discuss CM use with their conventional HCP?**							
	Very important	Quite important	Little important	Not important	No opinion		
	887 (60%)	328 (22%)	101 (7%)	26 (2%)	103 (7%)		41 (3%)
**How often do you motivate patients to discuss their CM use with their conventional HCP?**							
	Always	Sometimes	Never	Other ^e^			
	730 (49%)	401 (27%)	134 (9%)	189 (13%)			32 (2%)

Percentages may add to less or more than 100% due to rounding

^a^ E.g. contact through patient

^b^ E.g. via a secured digital environment or using multiple methods

^c^ E.g. relevant information depending on situation

^d^ E.g. situation-dependent (patient, symptoms, HCP)

^e^ E.g. patients’ own decision, patients are anxious to disclose CM use, lack of openness conventional HCP, situation-dependent

### Predictors of interprofessional contact

The explorative, multivariate logistic regression model shows three significant predictors of interprofessional contact with conventional HCPs as reported by CM practitioners (see Table 3). CM practitioners with more years of experience in treating patients with cancer were significantly more likely to have contact with conventional HCPs (OR = 1.05, 95% CI 1.04–1.06, *p* < .001), although the effect was small. Compared to CM practitioners who experience conventional HCPs as not being open to communication with them, CM practitioners who experience conventional HCPs as open to communication are significantly more likely to have interprofessional contact (OR = 8.12, 95% CI 5.12–12.86, *p* < .001). This also applies to CM practitioners who gave other answers (e.g. experienced openness of HCPs is situation-dependent), who are more likely to have contact with conventional HCPs compared to CM practitioners who experience conventional HCPs as not open (OR = 2.54, 95% CI 1.82–3.54, *p* < .001). CM practitioners who have no opinion on the experienced openness of HCPs are significantly less likely to have interprofessional contact with conventional HCPs compared to CM practitioners who experience HCPs as not open to communication (OR = 0.66, 95% CI 0.47–0.92, *p* < .05). CM practitioners who consider patient disclosure of CM use to their conventional HCP quite or little important are less likely to have contact with conventional HCPs of the patient compared to CM practitioners who consider patient disclosure of CM use very important (OR = 0.70, 95%CI 0.51–0.96, *p* < .01/OR = 0.39, 95%CI 0.23–0.68, *p* < .001).

**Table 3:** Multivariate logistic regression predictors for interprofessional contact (IPC) (*n* = 1186)

Predictor	B	S.E.	OR	95% CI
Sex (female)	-0.29	0.18	0.75	0.53–1.05
Age	0.02	0.01	1.02	1.00-1.04
Years of experience with cancer patients	0.05***	0.01	1.05	1.04–1.06
Monthly number of cancer patients visiting	0.03	0.02	1.03	1.00-1.06
Experienced openness of conventional HCP to IPC (Most HCP are not open to IPC)				
Most HCP are open to IPC (1)	2.09***	0.24	8.12	5.12–12.86
No opinion (2)	-0.42*	0.17	0.66	0.47–0.92
Other (3)	0.93***	0.17	2.54	1.82–3.54
Perceived importance of patient disclosure of CM use (Very important)				
Quite important (1)	-0.36*	0.16	0.70	0.51–0.96
Little important (2)	-0.94***	0.28	0.39	0.23–0.68
Not important (3)	-0.88	0.52	0.41	0.15–1.15
No opinion (4)	-0.39	0.30	0.68	0.38–1.21
*Constant*	-1.84	0.54		

*Note: R*^*2*^ *= 0.28 (Nagelkerke). Model χ2 = 276.59, p* < .001

*p < .05, **p < .01, ***p < .001

## Discussion

This study examined the experiences of CM practitioners with contact with conventional HCPs in oncology and the importance CM practitioners attach to patient disclosure of CM use to their conventional HCP. Potential predictors for interprofessional contact were explored. In total, 40% of the surveyed CM practitioners (*n* = 1486) indicated that they occasionally or frequently have contact with conventional HCPs of patients with cancer. The emergence of interprofessional contact seems to be mainly predicted by the extent to which CM practitioners experience conventional HCPs to be open to interprofessional communication. Most CM practitioners (82%) consider it important that patients with cancer disclose CM use to their conventional HCP and motivate their patients to disclose CM use.

In a previous survey, 18% of CM practitioners reported to have previously communicated with conventional HCPs [6]. The surveyed CM practitioners in the current study reported a much higher prevalence of previous contact with conventional HCPs, which might be explained by the frequent use (51%) of CM by patients with cancer [1]. The study results indicate that the CM practitioner is mostly the initiator of contact by reporting the treatment plan or treatment progress. The study of Schiff et al. [6] showed that most physicians and CM practitioners feel that the CM practitioner should initiate interprofessional communication.

Only a minority of the surveyed CM practitioners experienced conventional HCPs to be open to communication with them. This perceived lack of openness is in line with the reported skepticism towards and lack of knowledge on complementary medicine among conventional HCPs in oncology [13, 14]. However, since conventional HCPs were not surveyed in the current study, our findings do not reflect the actual openness of conventional HCPs to communication with CM practitioners. Previous studies showed that conventional HCPs find interprofessional communication less important [6] and are less supportive of opportunities to improve interprofessional communication when compared to CM practitioners [8]. Nurses were more supportive than medical doctors [8], implying that nurses could play a pivotal role in bridging the communication gap between conventional HCPs and CM practitioners.

A notable finding is that almost one-third of the surveyed CM practitioners reported having no opinion on their experience of openness of conventional HCPs to communication. Additionally, it was shown that these CM practitioners were significantly less likely to have contact with conventional HCPs compared to CM practitioners who experienced conventional HCPs as not open to communication. This could imply that these CM practitioners did not consider interprofessional contact relevant. The relevance of interprofessional contact between CM practitioners and conventional HCPs is situation dependent, e.g. in the case of cancer survivors who have completed treatment. Another possibility is that CM practitioners who indicated to have no opinion on the openness of conventional HCPs, have treated few cancer patients yet, making them unable to properly evaluate this topic. Indeed, the results showed that years of experience in treating patients of the CM practitioner was significantly associated with contact with conventional HCPs.

The role of CM practitioners in the patient disclosure of CM use to their HCP is an understudied topic in existing literature. The present study shows that a large majority of CM practitioners attach importance to patient disclosure of CM use and motivate their patients to discuss CM use with their conventional HCPs. The importance a CM practitioner attaches to patient disclosure of CM use to their conventional HCP can reflect how relevant they consider it that the conventional HCP is informed. Indeed, the results of this study showed that perceived importance of patient disclosure of CM use predicts whether a CM provider has contact with conventional HCPs.

CM practitioners highlighting the importance and encouraging a patient to discuss CM use could facilitate patient disclosure of CM use, which is reportedly hindered by a lack of inquiry by the healthcare provider, anticipation of disapproval by the healthcare provider or the perception that disclosing CM use is not relevant or patient’s [9, 15]. In the current study, experience with patients being anxious to disclose CM use to their conventional HCP was also reported in open-ended questions by the surveyed CM practitioners.

The specific situations in which contact between CM practitioners and conventional HCPs is relevant should be explored in a follow-up study. Nonetheless, it is important for HCPs to be aware of patient CM use since it can provide valuable medical information about the patient and their (unsolved) complaints. In addition, complementary medicine use may indicate dissatisfaction with conventional care [1]. Patients are often given the responsibility of informing the conventional HCP on their CM use. It is questionable whether patients should bear this responsibility, especially when it concerns the safety of combining CM with conventional anticancer treatment. For optimal monitoring of the health and safety of patients with cancer, there should be open communication about CM use between all parties involved: conventional HCPs, CM practitioners and the patient. This will prevent the disappearance of valuable medical information in the metaphorical “Bermuda Triangle” between the three parties [6].

### Strengths and limitations

This study is, to the best of our knowledge, the first to describe CM practitioners’ experiences with contact with conventional HCPs in oncology. To overcome sampling bias and include different types of CM practitioners, we approached an umbrella quality register. Although the average response rate among members of actively approached professional organizations was low (9%), the total sample size is large enough to outline the experiences of CM practitioners with interprofessional contact. The 9% response rate might have resulted in bias, for instance complementary medicine practitioners more willing to communicate with conventional healthcare providers responded, resulting in an overestimation of interprofessional contact. Furthermore, some types of CM practitioners are overrepresented in the sample, such as acupuncturists, because their professional associations were directly approached for survey distribution. In addition, most participants were females with a high education level. Whether this is representative of the population of CM practitioners in the Netherlands is not clear because sufficient oversight is lacking. In a comparable survey conducted in an oncology setting in Norway, the CM practitioners visited by patients with cancer were also predominantly female [8]. The sex of a CM practitioner was no significant predictor for contact with conventional HCPs.

Some limitations are associated with the survey. The fact that proportionately many participants chose the ‘other’ category for multiple-choice questions could indicate that the existing answer options were not sufficient. Respondents who answered in the ‘other’ categories often mentioned that they could not provide an unequivocal answer to the question posed because it was situation dependent. For example, experienced openness varies by HCP, or the relevance of interprofessional contact varies by patient. In addition, it was possible to proceed with the next question without answering the previous question, resulting in missing values.

### Future studies

The current study only highlighted the perspective of CM practitioners on interprofessional contact. Future research should focus on the needs and desired roles of conventional HCPs and patients in the process of interprofessional contact. It is unclear how patients feel about their intermediary role between CM practitioners and HCPs. Given that interprofessional communication is often a non-routinized, unstructured process, the appropriate method, frequency and content of communication should be further explored. For instance, it could be explored amongst conventional healthcare providers what type of information about complementary medicine use of their patients is of relevance, such as indication, content or outcomes of treatment by the complementary medicine practitioner. In addition, the factors that determine the openness of HCPs as experienced by CM practitioners could be investigated more in depth, for example by means of interviews.

## Conclusions

To conclude, interprofessional contact with conventional HCPs occurs but is not a standard routine for most CM practitioners. More than one-third of the surveyed CM practitioners experienced conventional HCPs as not open to communication with them. The openness of conventional HCPs as experienced by CM practitioners appeared to significantly determine whether interprofessional contact occurs. Most CM practitioners considered patient disclosure of CM use to their conventional HCP to be important. Open communication about the topic of CM use between CM practitioners, conventional HCPs and patients prevents overlooking relevant medical information and facilitates optimal monitoring of the health condition and safety of patients with cancer.

### Electronic supplementary material

Below is the link to the electronic supplementary material.

Supplementary Material 1

Supplementary Material 2

## Data Availability

The datasets used and/or analysed during the current study are available from the corresponding author on reasonable request.

## Abbreviations

CM: Complementary medicine
HCP: Healthcare provider
IPC: Interprofessional contact
