# Supplementary material for: Interprofessional contact with conventional healthcare providers in oncology: a survey among complementary medicine practitioners
Source: BMC Complement Med Ther. 2024 Jul 26;24:285. doi: 10.1186/s12906-024-04563-6 (PMC11282773; doi:10.1186/s12906-024-04563-6)
Supplement: Supplementary file 1 — Supplementary Material 1 [file 12906_2024_4563_MOESM1_ESM.docx]

**Additional file 1 – Comprehensive overview of CM practitioners background characteristics**

**Table A1.** Professional associations that distributed the survey link and response rates

| **Original name in Dutch (abbreviation)** | **English translation** | **No. of members** | **Response rate (%) ^a^** |
| --- | --- | --- | --- |
| Register Beroepsbeoefenaren Complementaire Zorg (RBCZ) | Register for Complementary Medicine Professionals | 8858 | 2% (n=182) ^b^ |
| Artsen Vereniging Integrale Geneeskunde (AVIG) | Physicians Association Integrative Medicine | 255 | 7% (n=18) |
| Nederlandse Vereniging voor Acupunctuur (NVA) | Dutch Association for Acupuncture | 1080 | 16% (n=169) |
| Nederlandse Artsen Acupunctuur Vereniging (NAAV) | Dutch Physicians Acupuncture Association | 122 | 5% (n=6) |
| Wetenschappelijke Artsen Vereniging voor Acupunctuur Nederland (WAVAN) | Scientific Doctors' Association for Acupuncture the Netherlands | 58 | 12% (n=7) |
| Netwerk Massage bij Kanker | Network Massage for Cancer | 110 | 4% (n=4) |
| Nederlandse Vereniging voor Traditionele Chinese Geneeskunde (Zhong) | Dutch Association for Traditional Chinese Medicine | 840 | 15% (n=54) |

Note: participants can be member of more than one professional association

^a^  Numbers include CM practitioners that indicated to not treat cancer patients

^b^ 1125 participants reported membership of one of the 24 professional associations attached to the umbrella register

**Table A2.** Answers Q7: Which types of cancer did patients who visit you have/had? (N=1486)

| **Answer** | ***n*** |
| --- | --- |
| Specific type(s) of cancer (see Figure A2) | 1106 |
| Diverse types of cancer | 216 |
| Other ^a^ | 12 |
| Missing | 152 |

^a^ E.g. ‘metastized cancer’, ‘cancer survivors’ or ‘irrelevant’

**Figure A2.** Number of CM practitioners reporting cancer type of patients they are visited by (n=1106).

Note: participants could report multiple answer options

**Table A3.** Number of CM practitioners reporting complementary therapy provided to patients with cancer (N=1482)

| **Complementary therapy** | **N** |
| --- | --- |
| **Manipulative and body-based therapies (total)**  Massage  Acupuncture  Shiatsu  Lymph drainage  Haptotherapy  Craniosacral therapy  Kinesiology  Fascia or triggerpoint therapy  Osteopathy  Other manual therapies | **1227**  479  310  73  62  46  39  38  27  12  10 |
| **Mind-body therapies (total)**  Relaxation exercises  Mindfulness  Hypnotherapy  Yoga  Art therapy  Music therapy  Body stress release  Breathing exercises  Qigong  Other mind-body interventions | **1019**  418  297  142  51  49  21  17  15  15  11 |
| **Lifestyle therapies (total)**  Lifestyle counseling  Dietary counseling  Exercise or movement advice | **984**  439  340  205 |
| **Psychological therapies (total)**  Psychotherapy  Body-focused psychotherapy  Psychosocial therapy  Coaching or counseling  Systemic therapy | **444**  203  68  67  58  48 |
| **Biological-based therapies (total)**  Supplements, herbs, plants  Aromatherapy | **342**  277  65 |
| **Energy therapies (total)**  Energetic therapy  Therapeutic or healing touch  Bioresonance | **135**  57  56  22 |
| **Whole medical systems (total)**  Homeopathy  Antroposofic medicine  Naturopathy  Mesology  Ayurveda  Other medical systems | **123**  86  11  8  7  6  5 |
| **Other interventions (total)** | **54** |

Note: participants can provide multiple complementary therapies

**Table A3.** Number of complementary practitioners reporting to treat symptom (n=1481)

| **Symptom** | **N** |
| --- | --- |
| Fatigue ^1^ | 1240 |
| Anxiety | 1012 |
| Sleeping problems | 944 |
| Depression | 874 |
| Concentration problems | 684 |
| Neuropathy | 657 |
| Psychosocial problems | 622 |
| Coping problems | 542 |
| Headache | 526 |
| Nausea | 519 |
| Stool problems | 516 |
| Muscle pain | 515 |
| Joint pain | 453 |
| Lack of appetite | 383 |
| Dyspnea | 363 |
| Vomiting | 233 |
| Drowsiness | 188 |
| Swallowing | 184 |
| Other ^2^ | 291 |

^1^ Merged with ‘lack of energy’ category due to magnitude of overlap (79%)

^2^ Mainly symptoms related to (physical) pain and psychological symptoms (emotions, mental health, mood).
