# Supplementary material for: Interprofessional contact with conventional healthcare providers in oncology: a survey among complementary medicine practitioners
Source: BMC Complement Med Ther. 2024 Jul 26;24:285. doi: 10.1186/s12906-024-04563-6 (PMC11282773; doi:10.1186/s12906-024-04563-6)
Supplement: Supplementary file 2 — Supplementary Material 2 [file 12906_2024_4563_MOESM2_ESM.docx]

**Additional file 2 - Survey**

1. What is your age?

…. Years

2. What is your sex?

- Male
- Female
- Other

3. What is your education?

……

4. Of which professional association are you a member?

……..

5. Do you see patients who have/had cancer?

- Yes
- No (End of survey)

6. We would like to know which therapy you offer to patients who have/had cancer. In addition, we would like to know if you followed additional education for oncology.

- Aroma care
- Acupuncture
- Art therapy
- Movement therapy
- Dietary advice
- Haptotherapy
- Hypnotherapy
- Lifestyle advice
- Massage
- Mindfulness
- Music therapy
- Edema therapy
- Relaxation exercises
- Osteopathy
- Therapeutic touch
- Nutritional supplements
- Yoga
- Other, namely……

7. Which type(s) of cancer did patients who visit you have/had?

……………………………………….

8. Which complaint(s) of patients who have/had cancer do you treat?

- Anxiety complaints
- Shortness of breath
- Vomiting
- Problems with concentration
- Coping
- Depression complaints
- Lack of appetite
- Lack of energy
- Joint pain
- Headache
- Nausea
- Neuropathy
- Problems with bowel movement
- Psychosocial problems
- Sleeping problems
- Swallowing problems
- Drowsiness
- Muscle pain
- Fatigue
- Other, namely……

9. How many years of experience do you have in providing therapy to patients who have/had cancer?

……………………………………….

10. How many patients who have/had cancer do you provide therapy to per month?

……………………………………….

11. When you provide therapy to patients who have/had cancer, how often do you averagely have contact with conventional healthcare providers (doctor or nurse) who treat or treated the patient?

- Never
- Once at the beginning of complementary treatment
- Once during complementary treatment
- Multiple times during complementary treatment
- At the end of complementary treatment
- Other, namely……

12. Which communication method do you use when communicating with a conventional healthcare provider who treat or treated the patient?

- Electronical medical record
- Letter
- Email
- Phone
- Face to face
- Other, namely……

13. What do you discuss with a conventional healthcare provider who treat or treated the patient?

……………………………………….

14. How do you experience the openness of conventional healthcare providers (doctors and nurses) who treat or treated patients with cancer towards communication about complementary medicine with you?

- Most doctors/nurses are open for communication
- Most doctors/nurses are not open for communication
- No opinion
- Other, namely……

15. How important do you think it is that your patients that have/had cancer and visit your practice, discuss their complementary medicine use with conventional healthcare providers (doctors and nurses)?

- Not important
- Little bit important
- Important
- Very important
- No opinion

16. How often do you motivate patients who have/had cancer and visit your practice, to discuss their complementary medicine use with conventional healthcare providers (doctors and nurses)?

- Always
- Sometimes
- Not at all
- Other, namely……

17. How do patients who have/had cancer and visit your practice get to visit you?

- These patients are (almost) always referred to me by an HCP
- There patients are (almost) never referred to me
- These patients are (almost) always referred to me by an HCP or get to me without referral
- Other, namely….
